# A pilot study to assess the utility of a freely downloadable mobile application simulator for undergraduate clinical skills training: a single-blinded, randomised controlled trial

**DOI:** 10.1186/s12909-017-1085-y

**Published:** 2017-12-11

**Authors:** Richard D. Bartlett, Dina Radenkovic, Stefan Mitrasinovic, Andrew Cole, Iva Pavkovic, Peyton Cheong Phey Denn, Mahrukh Hussain, Magdalena Kogler, Natalia Koutsopodioti, Wasima Uddin, Ivan Beckley, Hana Abubakar, Deborah Gill, Daron Smith

**Affiliations:** 1 UCL Medical School, University College London, London, UK; 2 UCL School of Pharmacy, University College London, London, UK; 3 Academic Centre for Medical Education, University College London, London, UK; 4 Department of Urology, University College London Hospital NHS Foundation Trust, London, UK

**Keywords:** Simulation, Touch surgery, Mobile application simulator, Virtual reality, Medical education, Clinical skills training, Medical students, Medical assessment, Objective structured clinical examination, Catheterization

## Abstract

**Background:**

Medical simulators offer an invaluable educational resource for medical trainees. However, owing to cost and portability restrictions, they have traditionally been limited to simulation centres. With the advent of sophisticated mobile technology, simulators have become cheaper and more accessible. *Touch Surgery* is one such freely downloadable mobile application simulator (MAS) used by over one million healthcare professionals worldwide. Nevertheless, to date, it has never been formally validated as an adjunct in undergraduate medical education.

**Methods:**

Medical students in the final 3 years of their programme were recruited and randomised to one of three revision interventions: 1) no formal revision resources, 2) traditional revision resources, or 3) MAS. Students completed pre-test questionnaires and were then assessed on their ability to complete an undisclosed male urinary catheterisation scenario. Following a one-hour quarantined revision period, all students repeated the scenario. Both attempts were scored by allocation-blinded examiners against an objective 46-point mark scheme.

**Results:**

A total of 27 medical students were randomised (*n* = 9 per group). Mean scores improved between baseline and post-revision attempts by 8.7% (*p* = 0.003), 19.8% (*p* = 0.0001), and 15.9% (*p* = 0.001) for no resources, traditional resources, and MAS, respectively. However, when comparing mean score improvements between groups there were no significant differences.

**Conclusions:**

Mobile simulators offer an unconventional, yet potentially useful adjunct to enhance undergraduate clinical skills education. Our results indicate that MAS’s perform comparably to current gold-standard revision resources; however, they may confer significant advantages in terms of cost-effectiveness and practice flexibility.

**Trial registration:**

Not applicable.

**Electronic supplementary material:**

The online version of this article (10.1186/s12909-017-1085-y) contains supplementary material, which is available to authorized users.

## Background

Medical simulators offer a potentially invaluable educational resource for medical trainees. They allow procedures to be practiced in a formative environment and permit procedure rehearsal with minimal risk to patient safety. Furthermore, they facilitate the step-by-step breakdown of complex psychomotor tasks, and allow for consistent procedure replicability. To date, medical simulators have been used in a range of healthcare education settings [1]. Their most notable success has been in post-graduate surgical education, where they have been used to teach surgical trainees a range of procedures. These range from fracture fixation and shoulder arthroscopy in the context of orthopaedics [2, 3], to the unique psychomotor skills required for laparoscopic and endoscopic procedures in general surgery [4–7]. However, despite their wide-spread adoption into postgraduate training, traditional simulators have had limited uptake in the undergraduate setting. This may be because they are typically expensive and immobile, and consequently their use is limited to designated simulation training centres [8]. Student access to such centres is often limited by time and cost, and this may adversely affect uptake and skill acquisition [9].

Nevertheless, with the advent of increasingly sophisticated mobile technology, simulators suitable for teaching have become cheaper and more portable [10, 11]. One simulator which has been at the vanguard of this transition is the *Touch Surgery* mobile application. This is a free app-based simulator downloadable from the Google Play and iOS stores, which comprises a catalogue of several hundred operations and practical procedures. All procedures are developed in combination with, and reviewed by, procedure-specific experts [12]. The app constructs a rendered 3-dimensional virtual reality environment, and then guides users through every stage of each procedure using touch-screen motion gestures. In turn, this allows users to rehearse the steps of the procedure: a technique known as ‘cognitive task analysis’ [13, 14]. *Touch Surgery* has previously been validated for intramedullary femoral nailing [15], yet, to date, its validity as a training tool in more commonly performed ward-based clinical skills and undergraduate level procedures remains unknown.

As such, the primary aim of this pilot study was to evaluate whether mobile application simulators (MAS’s) are a useful alternative to traditional educational approaches for medical undergraduates revising routine, ward-based clinical skills (e.g. male urinary catheterisation). Secondary aims included: 1) to assess the confidence of medical students in performing core clinical skills procedures, and 2) to determine whether self-assessed confidence correlated with objectively scored performance.

## Methods

### Study design

**Fig. 1:**
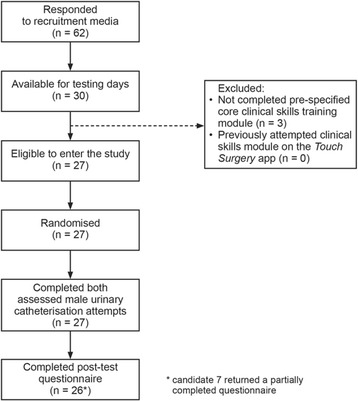
Overview of trial recruitment, randomisation and follow-up. Note: candidate 7 was included in the main body of the analysis, and only their results for the post-test questionnaire were excluded

We piloted a pragmatic single-blinded, randomised controlled study design to evaluate the effectiveness of a freely available and widely used MAS. A comparative three-arm trial design was used, allowing us to compare the following groups: 1) no formal teaching intervention, 2) traditional gold-standard learning resources, and 3) MAS (Fig. 1).

### Recruitment

Clinical medical students in years 4–6 of a six-year programme were recruited from a single London medical school. Students were recruited using a combination of emails, ‘shout-outs’ in lectures, flyers, and social media. To be eligible to enter the study, students needed to have previously completed the ‘Core Clinical Skills Module’ delivered as part of the year 4 curriculum – this includes foundation training in core clinical competencies such as venepuncture, cannulation, arterial blood gases, preparation of injections, suturing, and male urinary catheterisation. Students were told in advance that they would be required to perform a ward-based clinical skill under OSCE style exam conditions as part of the study, however, the nature of the procedure was not disclosed. This fitted with our aim of trying to determine how the MAS would perform in a real-life scenario. Similarly, students were told not to specifically revise in advance of the study, and this was in order to ascertain a representative assessment of baseline day-to-day competence.

Students were informed that they were ineligible to enter the study if they had previously attempted any of the clinical skills modules on the MAS. Qualified doctors (> Foundation Year 2 grade and with a previous placement in urology) were recruited from local teaching hospitals to act as examiners.

### Randomisation

Recruited students were allocated a unique candidate number and then split by the date of their availability (2 test dates were available). To minimise bias, students were stratified by year group (4, 5, or 6) and then randomised by RDB using their unique candidate number to one of the three intervention groups by an online computer-based random number generator (https://www.random.org/). Following randomisation, students in each group were evenly divided between five examiners, such that each examiner was apportioned with an approximately equal number of students from each of the intervention groups.

### Testing protocol

Testing took place over two pre-planned testing days at a clinical skills simulation centre. Participants consent to continue with the study was initially confirmed and then a pre-test questionnaire was completed. This utilised a 10-point Likert scale (scored 1–10) to ascertain self-assessed student confidence in performing each of the core competencies taught as part of the Core Clinical Skills Module. Under simulated exam conditions, students were then assessed on their ability to perform a male urinary catheterisation scenario on a manikin. Male urinary catheterisation was selected for its complex multi-step nature, and also for its availability on the MAS procedure library at the time of testing. Assessment was designed to be pragmatic in nature, and hence aimed to replicate what a student might reasonably be expected to perform as a junior doctor on a medical ward. As such, not only was knowledge of the steps involved in the procedure tested, but also the ability to consent, independently gather equipment, maintain aseptic technique, and appropriately document information in the notes. Whilst it was not possible to blind students to group allocation, examiners did not know which revision curriculum students were allocated to. Examiners scored students against a 46-point gold-standard objective structured clinical examination (OSCE) style mark scheme, which was developed with input from author DS, a practicing consultant urologist. A total of 15 min was permitted to complete the scenario. Copies of the candidate vignette, 46-point mark scheme, and mock patient notes can be found in Additional file 1, Additional file 2 and Additional file 3, respectively.

After the initial baseline assessment, students were quarantined for 1 h. The control group did not have access to any revision resources between their first and second procedure attempts. This group served to measure any improvement that may have occurred by students simply repeating the same procedure (repetition learning). Students in the traditional resources group had access to the educational material that would normally be available to use whilst undertaking revision at a clinical skills centre: a sample video on how to perform the procedure, lecture slides, a model mark scheme (independent of the one developed specifically for this study), manikins, and male urinary catheterisation equipment. Those allocated to the MAS group had access to the male urinary catheterisation modules available on the mobile application only (i.e. they did not have access to any of the resources available to the traditional resources group). All students were individually quarantined for the duration of the 1 h period between their first and second assessed male urinary catheterisation attempts.

**Fig. 2:**
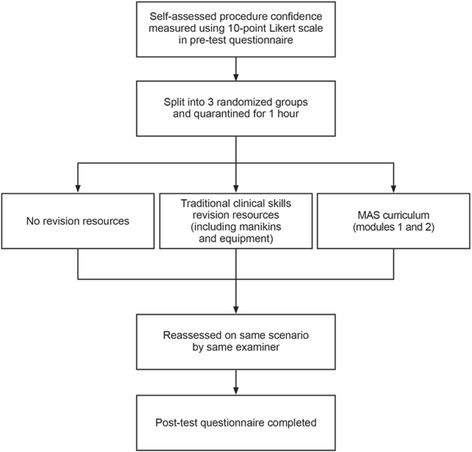
Flow-process of the testing procedure. Students were split evenly between the three groups

Following the quarantine period, all students were re-assessed. Examiners scored the same students for both assessed attempts in order to reduce inter-examiner bias, and, as with the first assessed attempt, the only communication permitted between examiner and student was that which was pre-determined by the mark scheme. The same mark scheme was used to assess both attempts. At no point were examiners aware of which group students had been allocated to. Students then completed a post-test questionnaire. The overall testing process is summarised in Fig. 2.

### Statistical analysis

IBM SPSS Statistics 22.0 (IBM Inc. Armonk, NY) was used for all statistical analysis. Statistical tests were assumed to be significant at the 5% level. The Shapiro-Wilk test was used to assess the normality of data. Where data was found to be normally distributed, parametric tests were used. Where appropriate, Levene’s test statistic was used to assess for equality of variance. One-way ANOVA was used to compare mean values of multiple groups and paired t-test was used to compare the pre- and post-scores of individual groups. Pearson’s correlation co-efficient was used to assess student self-assessed confidence against examiner score, and multiple linear regression was used to assess specific factors that might have predicted OSCE performance. A power calculation using data from a previous summative OSCE in male catheterisation was also performed prior to recruitment. Based on this limited data, a sample size was calculated to be *n* = 10 per group to detect a 20% change in raw mark with α = 0.05 and power = 80%.

## Results

A total of 62 students responded to recruitment media, of which 30 were available for the scheduled testing dates. No students had previously attempted the clinical skills modules on the MAS, however, *n* = 3 were unexpectedly excluded from testing because they had not completed the pre-specified Core Clinical Skills Module (despite this being explicitly specified in all recruitment media). As such, a total of 27 students were entered into the study, and, of these, 17 (63%) were in year 4 and 10 (37%) were in year 5. All students who entered the study went on to complete it (*n* = 27), however, one post-test questionnaire was returned but not fully completed (candidate 7; traditional educational resources group). Construct validity was tested by assessing the three year 4 students who had not completed the pre-specified Core Clinical Skills Module using the same scenario (mean score = 12.3 / 46 ± 9.29 SD). The baseline characteristics for each group are summarised in Table 1.

**Table 1:** Baseline characteristics of each intervention group. Displays frequencies for the number of students from each year in each intervention group, along with mean baseline OSCE score ±1 SD

	No resources group	Traditional resources group	MAS group
Year 4	6 (66.7%)	5 (55.6%)	6 (66.7%)
Year 5	3 (33.3%)	4 (44.4%)	3 (33.3%)
Total	9 (100%)	9 (100%)	9 (100%)
Mean baseline OSCE score	28.7 ± 6.84 (62.3 ± 14.8%)	27.0 ± 5.94 (58.6 ± 12.9%)	27.6 ± 5.36 (60.0 ± 11.7%)

**Fig. 3:**
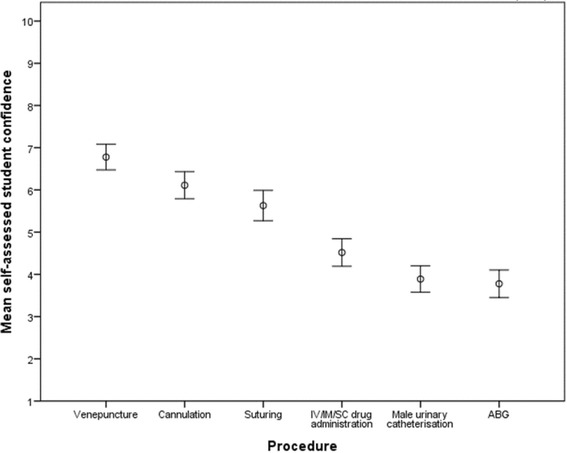
Mean self-assessed student confidence in core clinical skills procedures prior to baseline assessment, as assessed by a 10-point Likert scale. Error bars indicate ±1 SEM (*n* = 27). Abbreviations: ABG - arterial blood gas; IV - intravenous; IM - intramuscular; SC – subcutaneous

**Fig. 4:**
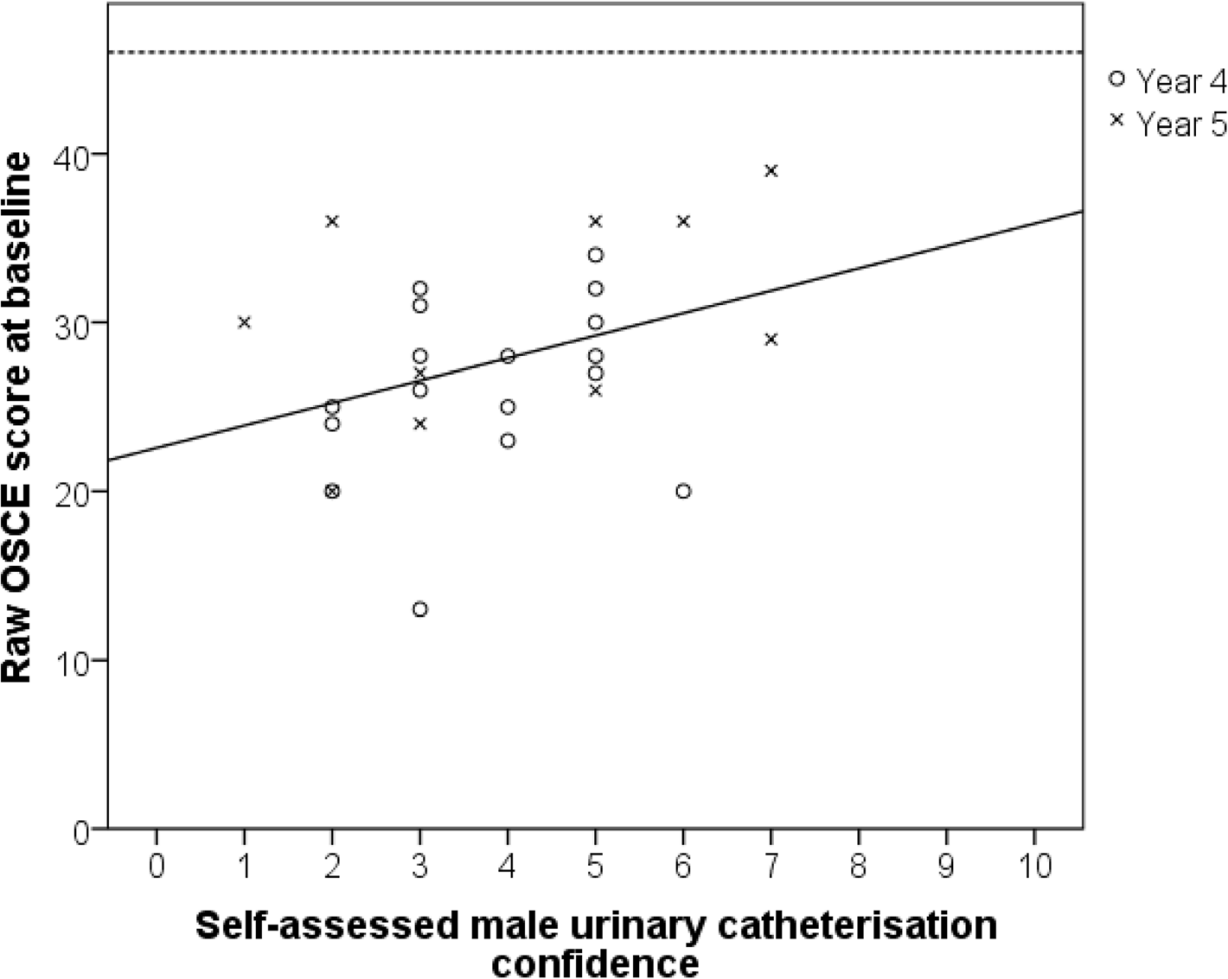
Correlation between self-assessed student procedure confidence and examiner assessed OSCE performance. Solid line displays line of best fit (Pearson co-efficient = 0.367, *p* = 0.06); dashed horizontal line represents maximum achievable score of 46 marks (n = 27)

Pre-test questionnaires revealed that student self-assessed confidence varied considerably between clinical skill (Fig. 3). Arterial blood gases (ABGs) were the procedure that students felt least confident in performing (mean Likert score 3.7 / 10), and this was closely followed by male urinary catheterisation (mean Likert score 3.88 / 10). Overall, self-assessed student confidence was found to poorly correlate with examiner assessed performance in male urinary catheterisation (Pearson’s co-efficient 0.367, *p* = 0.06, Fig. 4). However, multiple linear regression analysis revealed that self-assessed student confidence was a better predictor of OSCE performance than year group or examiner allocation (standardised beta coefficients: 0.357 vs. 0.298 and 0.156, respectively). Indeed, pre-test self-assessed male urinary catheterisation confidence was not found to vary significantly by year group (mean Likert scores: year 4 = 3.7 / 10, year 5 = 4.1 / 10, *p* = 0.614).

**Fig. 5:**
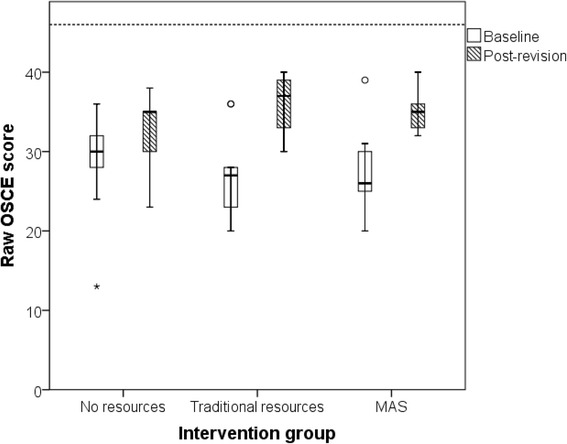
Distribution of raw OSCE scores at baseline and after 1-h spent with specified revision resources. Box and whisker plots display median and IQR values. Circular data points indicate outliers; asterisk indicates severe outlier. Dashed horizontal line depicts maximum achievable score of 46 marks (*n* = 9 per group). All students included in main analysis

The performance of all groups improved between first (baseline) and second (post-revision) male urinary catheterisation attempts (Fig. 5). The magnitude of score improvement was greatest in the traditional resources group, followed by MAS and then control (Table 2). However, when assessed by one-way ANOVA, the differences between the three groups was not statistically significant (*p* = 0.059). Year 5 students had higher baseline OSCE scores than year 4 students, but this difference was not statistically significant (mean difference = 4.0, *p* = 0.083).

**Table 2:** Changes between baseline and post-revision OSCE test scores. Uncorrected *p*-values derived from the results of paired t-tests. *P*-value required to achieve 5% significance level when corrected for multiple comparisons = *p* < 0.0167

	Mean baseline OSCE score (maximum / 46)	Mean post-revision OSCE score (maximum / 46)	Mean score change from baseline (95% CI)	Uncorrected p-value
No resources (n = 9)	28.7 (62.3%)	32.7 (71.1%)	4.0 (1.8–6.2) 8.7% (3.9% – 13.5%)	0.003
Traditional resources (n = 9)	27.0 (58.7%)	36.1 (78.5%)	9.1 (4.7–13.5) 19.8% (10.2% - 29.3%)	0.001
MAS (n = 9)	27.6 (59.8%)	34.9 (75.7%)	7.3 (4.3–10.4) 15.9% (9.3% - 22.6%)	0.001

Post-test questionnaire data revealed that self-assessed male urinary catheterisation confidence improved across all groups following two attempts at the procedure, and confidence was non-significantly higher in the MAS group relative to that of control or traditional resources (7.3 vs. 6.3 vs. 5.9 / 10 on Likert scale, respectively). Post-test questionnaire data also revealed that students thought app-based revision would make a useful addition to the clinical skills curriculum (mean Likert score 7.6 / 10), even though the standard MBBS course already includes the use of gold-standard training resources (such as videos, models etc.) used by the traditional resources group. Similarly, students who had been allocated to the MAS group rated usefulness and user-friendliness highly (mean Likert scores 6.9 / 10 and 7.0 / 10, respectively).

## Discussion

This single-blinded, randomised controlled pilot study demonstrated that focussed periods of revision, be it traditional or otherwise, improved medical student performance in male urinary catheterisation. As might be expected, the raw marks of all three groups (baseline vs. post-revision) significantly improved. The greatest change was observed in the gold-standard educational resources group (using models and equipment), yet there were no statistically significant differences when comparing the mean score changes between groups.

We also observed that, despite formal training as part of the medical school curriculum, students had variable self-assessed confidence in performing a range of core clinical skills. Specifically, they felt least confident in performing arterial blood gases and male urinary catheterisation. However, overall, we found that self-assessed confidence was a poor correlator of objectively assessed performance.

The *Touch Surgery* MAS has previously been trialled in intramedullary femoral nailing [16], where, concordant with our results, the app was well-received by users owing to its easy to navigate graphics and user-friendly interface. Yet, to the best of our knowledge, this is the first time that a MAS has be trialled in the context of augmentative undergraduate clinical skills training. Similar to our findings, Karim et al. have showed that medical students have variable confidence in history taking, physical examination and procedural skills [17]. However, in contrast to work performed by Tomic et al. [18], we did not find that student year group (an indicator of experience) correlated with self-assessed male urinary catheterisation confidence. Our results also indicated that self-assessed procedure confidence was only a weak predictor of examined performance, and this was consistent with other work on a large cohort of 122 final year medical students by Chen et al. [19]. However, contrary to the work of Chen et al.*,* students in our study had a low level of baseline confidence in male urinary catheterisation. Of those surveyed by Chen et al., 71% stated they were happy to teach the Foley catheterisation, whilst we observed a mean Likert self-assessed confidence score of only 3.8 / 10.

In contrast to traditional educational resources, app-based revision may confer a number of unique advantages for medical students and junior practitioners. Where access to extensive skills centre revision resources is limited - for example, at satellite hospitals without dedicated clinical skills training centres - or at times when the student wishes to study independently, our results suggest that MAS-based revision may be superior to repetitive practice alone. As alluded to, the flexibility and accessibility of MAS’s are unrivalled when compared to traditional revision methods. Our data suggest that students found the MAS interface both useful and user-friendly, and students also agreed that MAS-based revision was likely be a useful adjunct for improving clinical skills training in the future. Overall, this means that MAS-based revision resources offer a desirable revision alternative when traditional training equipment or facilities are limited. Likewise, the freely available nature of MAS’s means that they may have additional economic benefits [20], both for the trainee and training provider [21, 22].

We hope that this pilot study will serve as a useful springboard for future research wishing to investigate how MAS’s can be utilised in the context of undergraduate medical education. With the increased digitalisation of healthcare and readily available nature of personal electronic devices, MAS’s have great potential as a learning tool for medical students. Building on this work, future studies should utilise larger sample sizes, recruit from a range of medical schools, and test a greater breadth of undergraduate practical procedures to increase the external validity of the results presented here. Only through further testing will it become clear whether MAS-based revision resources should be have place in shaping the future of medical school curricula.

### Study limitations

In order to ensure standardised baseline knowledge between the intervention groups, we recruited from a single medical school only. Therefore, although all medical students must complete some core clinical skills training to meet the GMC-mandated outcomes for graduation [23], this may limit the inferences which can be drawn for other medical student populations (particularly outside the UK or where graduation competencies differ). Further, given that our students were only recruited from years 4 and 5 of the programme (the trial dates clashed with year 6 elective placements), it is possible that student confidence and competence may significantly increase as students approach the point of graduation; confidence may also differ between male and female urinary catheterisation. The limited sample size of this pilot also meant that the study was underpowered to detect potentially subtle differences in mean score change between the intervention groups. Indeed, we hope that the data presented here will enable higher powered studies to be conducted in the future. Finally, although OSCE style assessments are widely-regarded as the gold-standard method of clinical skills assessment [24, 25], it must be remembered that they can never be truly representative of a hospital or ward environment [26].

## Conclusions

MAS’s may offer an unconventional, yet useful and convenient adjunct to clinical skills education for medical undergraduates. Our results indicate that performance of the MAS we tested was similar to current gold-standard educational resources, even for the revision of a complex clinical skill. Moreover, the use of mobile technology may resolve a number of the problems associated with access to dedicated clinical skills centres and the equipment required for independent revision. Future work should consider testing MAS’s with other common clinical skills and with larger and more diverse student populations. It may also be worthwhile to investigate the effects of long-term skill retention with spaced repetition and independent study. Finally, given the problems associated with clinical skills centre access at the junior doctor level, validation of MAS’s amongst workplace trainees for more advanced procedures (e.g. chest drains) may also be worthy of future exploration.

## Additional files

Additional file 1: Copy of the OSCE scenario vignette. (PPTX 85 kb)

Additional file 2: Copy of the marking criteria used by examiners. (PPTX 277 kb)

Additional file 3: Copy of the mock medical notes that candidates were required to complete. (PPTX 71 kb)
