# Supplementary figures and images for: A pilot study to assess the utility of a freely downloadable mobile application simulator for undergraduate clinical skills training: a single-blinded, randomised controlled trial
Source: BMC Med Educ. 2017 Dec 11;17:247. doi: 10.1186/s12909-017-1085-y (PMC5725819; doi:10.1186/s12909-017-1085-y)

## Slide 1
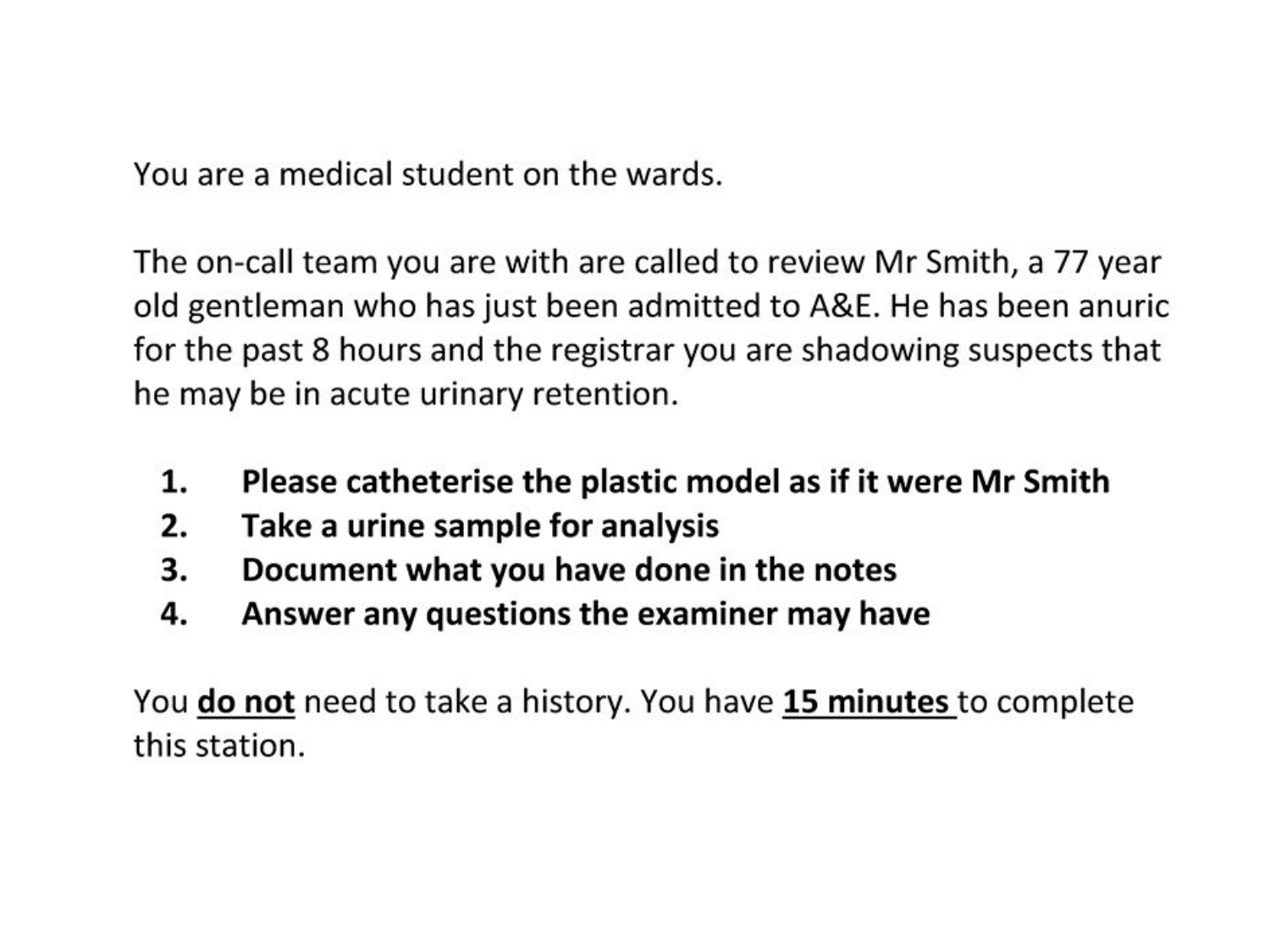

Supplement: Supplementary file 1 — Copy of the OSCE scenario vignette. (PPTX 85 kb) [file 12909_2017_1085_MOESM1_ESM.pptx]

## Slide 1
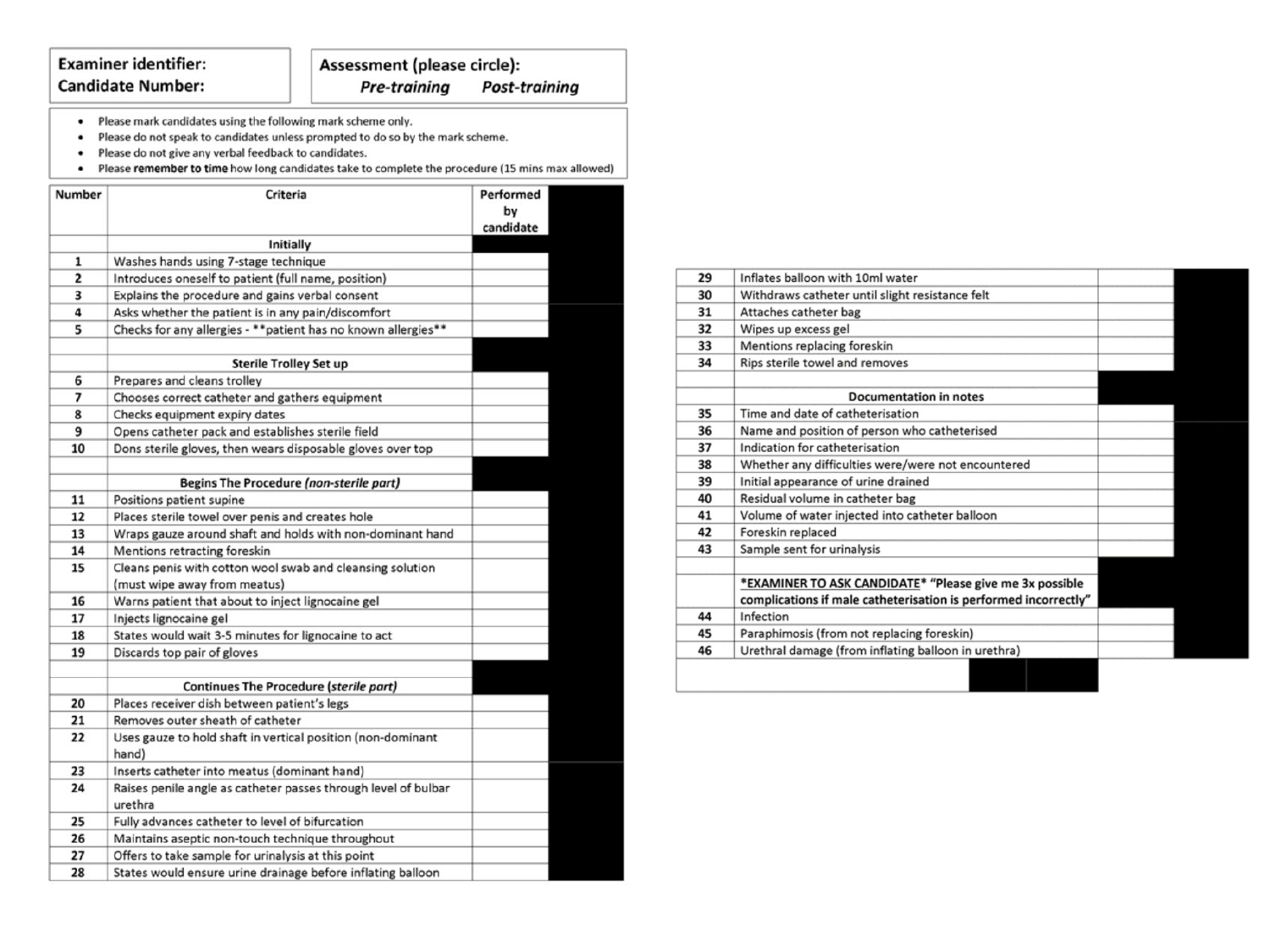

Supplement: Supplementary file 2 — Copy of the marking criteria used by examiners. (PPTX 277 kb) [file 12909_2017_1085_MOESM2_ESM.pptx]

## Slide 1
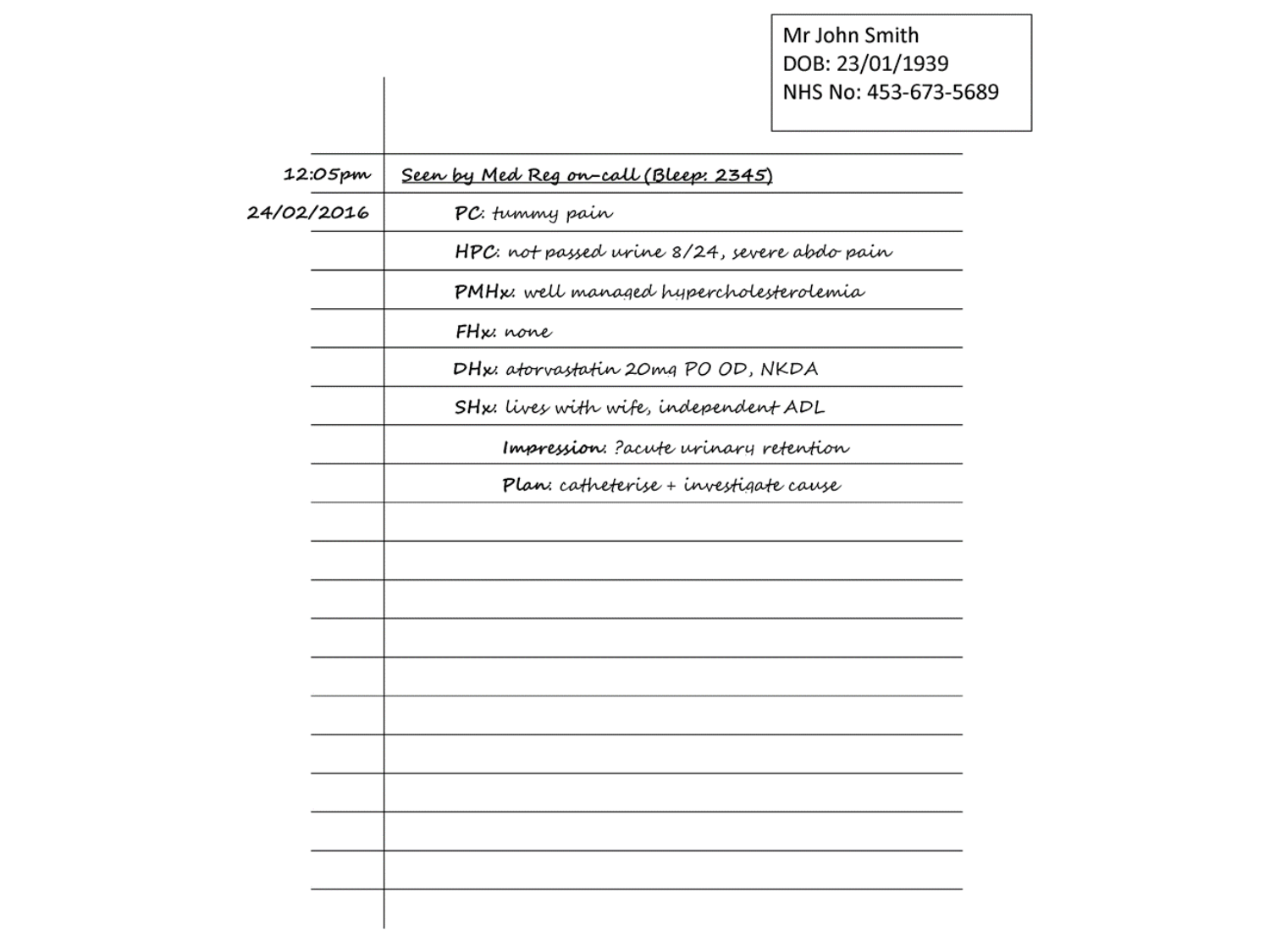

Supplement: Supplementary file 3 — Copy of the mock medical notes that candidates were required to complete. (PPTX 71 kb) [file 12909_2017_1085_MOESM3_ESM.pptx]
